# FABP4-dependent fatty acid oxidation-fueled mitochondrial ROS induces the mobilization of cellular iron and facilitates *Trypanosoma cruzi* proliferation in murine adipocytes

**DOI:** 10.1128/mbio.02180-25

**Published:** 2025-09-08

**Authors:** Kazunari Ishii, Yusuke Kurihara, Michinobu Yoshimura, Nirwana Fitriani Walenna, Akinori Shimizu, Ryo Ozuru, Kenji Hiromatsu

**Affiliations:** 1 Department of Microbiology & Immunology, Faculty of Medicine, Fukuoka University 12774, Fukuoka, Japan; Georgia Institute of Technology, Atlanta, Georgia, USA; California State University Fullerton, Fullerton, California, USA

**Keywords:** *Trypanosoma cruzi*, adipocyte, FABP4, fatty acid oxidation, mitochondria, ROS, labile iron pool

## Abstract

Fatty acid-binding protein 4 (FABP4) is a cytosolic lipid chaperone predominantly expressed in adipocytes. It has been shown that *Trypanosoma cruzi* targets adipose tissues and resides in adipocytes. However, how *T. cruzi* manipulates adipocytes to redirect nutrients for its benefit remains unknown. Here, we uncover the role of FABP4 in facilitating *T. cruzi* infection in murine 3T3-L1 adipocytes. We demonstrate that pharmacological or genetic inhibition of FABP4, carnitine palmitoyltransferase I (CPT-1), or fatty acid oxidation (FAO) abrogates the intracellular growth of *T. cruzi* in adipocytes. We also found that inhibiting FABP4, CPT-1, or FAO eliminates the infection-induced elevation of mitochondrial and cellular reactive oxygen species (ROS) in adipocytes. Furthermore, *T. cruzi* infection-induced elevation of ROS in adipocytes increased the cytosolic Fe^2+^, which fueled *T. cruzi* proliferation. The treatment with antioxidants such as ROS scavenger N-acetyl cysteine (NAC) or mitochondrial ROS inhibitors MitoQ increased the expression level of mRNA for Ferroportin and Ferritin, leading to the decrease in cytosolic Fe^2+^ and the intracellular growth inhibition of *T. cruzi* in adipocytes. The addition of ferrous sulfate reversed the FABP4 inhibitor or antioxidant-induced decrease in adipocyte parasite burden. Our results demonstrate that *T. cruzi* exploits host FABP4 to facilitate fatty acid oxidation and elevate cellular ROS, increasing the labile iron pool for the intracellular replication of *T. cruzi* in adipocytes. These results highlight the therapeutic possibility of host FABP4 as a drug target for *T. cruzi* infection.

**IMPORTANCE:**

Persistent infection with a protozoan parasite*, Trypanosoma cruzi,* causes Chagas disease. While it has been appreciated that adipose tissues are one of the sites of persistent infection, the mechanism of how the parasite survives in adipocytes remains to be established. Our study highlights FABP4, a key regulator of metabolic dysfunction and inflammation, as a therapeutic host target controlling *T. cruzi* infection in adipocytes. We uncover the importance of FABP4 for *T. cruzi* replication in mouse adipocytes through engagement with lipid droplet degradation and trafficking of liberated free fatty acids to the host cell’s mitochondria, which are utilized for fatty acid oxidation (FAO). *T. cruzi* infection-induced FAO fuels reactive oxygen species, and the subsequent iron mobilization accelerates parasite replication. These results shed light on the mechanisms of *T. cruzi* persistent infection in adipocytes, raising the possibility of host FABP4 as a drug target for *T. cruzi* infection.

## INTRODUCTION

Chronic infection with the obligate intracellular protozoan *Trypanosoma cruzi* (*T. cruzi*) can cause Chagas disease (1). *T. cruzi* infection favors tissues with high metabolic activity, such as the heart and adipose tissues (2). These tissues are characterized by significant fatty acid metabolism (3, 4). Previous studies have shown that *T. cruzi* targets adipose tissue and adipocytes (5, 6). It has been demonstrated that *T. cruzi* infects, induces lipolysis, and proliferates within adipocytes (7). Lipids stored in lipid droplets (LDs) of host cells serve as potential sources of fatty acids for parasites. It has been proposed that chronic *T. cruzi* infection in adipose tissues plays a crucial role in the parasite’s pathogenesis and persistence, involving mitochondrial stress responses (8). However, how *T. cruzi* benefits from host fatty acid metabolism and the molecular mechanisms behind persistent infection of adipocytes remain unclear. Using genome-scale functional screening, Caradonna et al. identified long-chain fatty acid oxidation as a vital process in mammalian host cells associated with intracellular *T. cruzi* growth (9). It remains to be determined whether *T. cruzi* amastigotes utilize fatty acid intermediates produced in host cells, mitochondrial oxidative pathways, or if they indirectly benefit from ATP production.

Although many pathogens are vulnerable to reactive oxygen species (ROS), some, such as *T. cruzi*, resist the oxidative environment (10, 11). Nuclear factor erythroid-derived 2-like 2 (NRF2) regulates antioxidant defenses, including the expression of heme-oxygenase-1 (HO-1). Paiva et al. found that several antioxidants, such as the NRF2 activator cobalt protoporphyrin (CoPP), reduced *T. cruzi* amastigote burden in macrophages, while pro-oxidants increased it (12). They also observed that lowering the intracellular labile iron pool decreased parasitism and that antioxidants enhanced the expression of ferritin and ferroportin in infected macrophages. However, whether oxidative stress encourages *T. cruzi* parasitism and growth in other cell types, such as adipocytes, remains unclear.

Fatty acid-binding protein 4 (FABP4) is a cytosolic lipid chaperone highly expressed in adipocytes and macrophages. It regulates lipid trafficking and cellular responses and is linked to metabolic and inflammatory pathways (13). It has been suggested that FABP4 actively facilitates the transport of fatty acids to specific organelles within the cell, such as the mitochondria or peroxisomes for β-oxidation, the nucleus for transcriptional regulation, trafficking, and membrane synthesis in the endoplasmic reticulum (ER), and storage as lipid droplets in the cytoplasm. FABP4 controls essential immuno-metabolic functions, contributes to adipose tissue inflammation and dysfunction, and links obesity and obesity-related disorders, such as atherosclerosis and insulin resistance (14). We previously reported that *Chlamydia pneumoniae* exploits FABP4 to facilitate fat mobilization and intracellular growth in murine adipocytes *in vitro* (15) and white adipose tissues *in vivo* (16). Recently, it has been reported that FABP4 is a key host factor involved in viral replication and lung pathology during coronavirus infection, highlighting its potential as a therapeutic target (17). However, the role of FABP4 in *T. cruzi* infection of adipocytes and adipose tissues remains to be determined.

Here, we reveal the crucial role of FABP4 in *T. cruzi* infection in mouse 3T3-L1 adipocytes. We found that *T. cruzi* uses host FABP4 to promote fatty acid oxidation and increase cellular ROS, raising the labile iron pool for the parasite’s intracellular replication in adipocytes.

## RESULTS

### The proliferation of *T. cruzi* increases in mature adipocytes and is linked to lipid droplet breakdown

**Fig 1:**
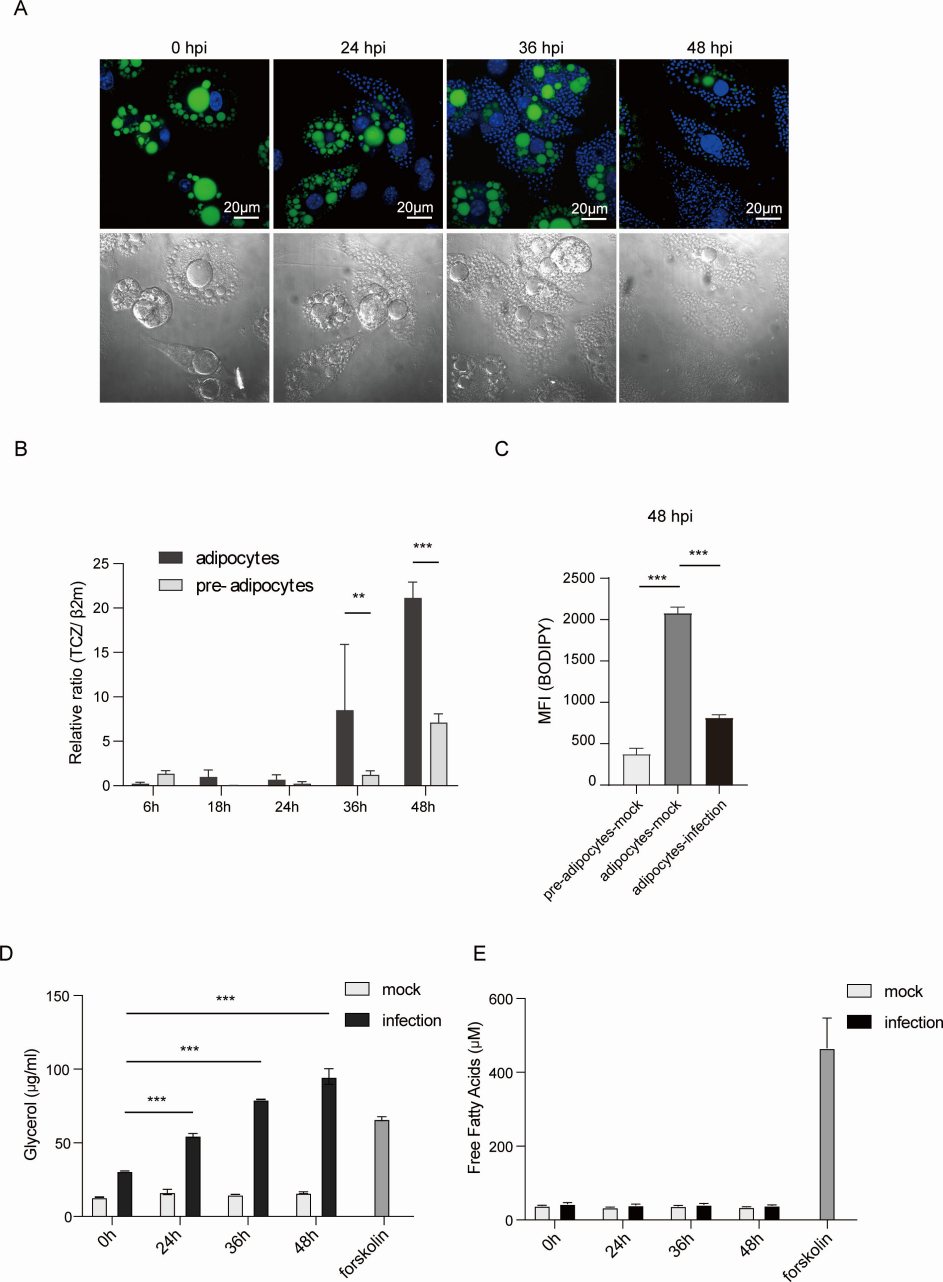
Proliferation of *T. cruzi* is enhanced in mature adipocytes and is associated with LD hydrolysis. (**A**) 3T3-L1 mature adipocytes were infected with *T. cruzi* MOI 10 for the indicated hours and examined for lipid droplets (BODIPY 493/503, green) and DNA (Hoechst33342, blue). DIC: differential interference contrast. Scale bar: 20 µm. (**B**) Quantitative genomic DNA PCR of *T. cruzi* in 3T3-L1 preadipocytes or mature adipocytes after infection with *T. cruzi*. (**C**) Mean fluorescence intensity (MFI) of BODIPY in 3T3-L1 pre-adipocytes or mature adipocytes after mock or *T. cruzi* infection at 48 hpi. *n* = 3 per group. (**D and E**) Secretion of glycerol (**D**) and free fatty acid (**E**) was measured in the cultured medium of 3T3-L1 adipocytes after *T. cruzi* infection (MOI of 10) or mock infection. A 4 h incubation with Forskolin (20 µM), an adenyl cyclase activator, was used as a positive control for lipolysis. The data are shown as mean ± SEM and are representative of at least three experiments. A two-way ANOVA with Sidak’s multiple comparison test (**B**) and a one-way ANOVA with Tukey’s multiple comparison test (C, D, and E) were used to analyze multiple groups. *P < 0.05, **P < 0.01, ***P < 0.001, n.s., not significant.

To elucidate how *T. cruzi* proliferates in 3T3-L1-differentiated adipocytes, we examined the lipid droplet (LD) fate of 3T3-L1 adipocytes after infection with *T. cruzi*. As shown in Fig. 1A, *T. cruzi* infection decreased LD content determined by BODIPY 493/503. With the increase in the number of amastigotes of *T. cruzi,* which are shown as Hoechst33342-positive small round materials in adipocytes, the size and contents of LD decreased. Quantitative genomic DNA PCR of *T. cruzi-specific* gene in 3T3-L1 preadipocytes or mature adipocytes at 48 hours post-infection (hpi) revealed that the proliferation of *T. cruzi* was enhanced in mature adipocytes compared to pre-adipocytes (Fig. 1B). *T. cruzi* infection-induced lipolysis was confirmed by BODIPY flow cytometry analysis of mature adipocytes infected with *T. cruzi* at 48 hpi (Fig. 1C), which corroborates the previous reports (18, 19). The activation of β-adrenergic receptors by norepinephrine triggers adipocyte lipolysis by stimulating adenylyl cyclase, which increases cyclic AMP levels to activate Protein kinase A (PKA). This results in the activation of hormone-sensitive lipase (HSL) (20). Forskolin, an adenyl cyclase activator, was used as a positive control for lipolysis (Fig. 1D and E). Notably, an apparent dissociation between glycerol release and free fatty acids (FFAs) release from *T. cruzi*-infected adipocytes (Fig. 1D and E) indicates that liberated FFAs are utilized intracellularly.

### Intracellular FABP4 protein levels, FABP4 secretion, and *Fabp4* mRNA expression in adipocytes during *T. cruzi* infection

**Fig 2:**
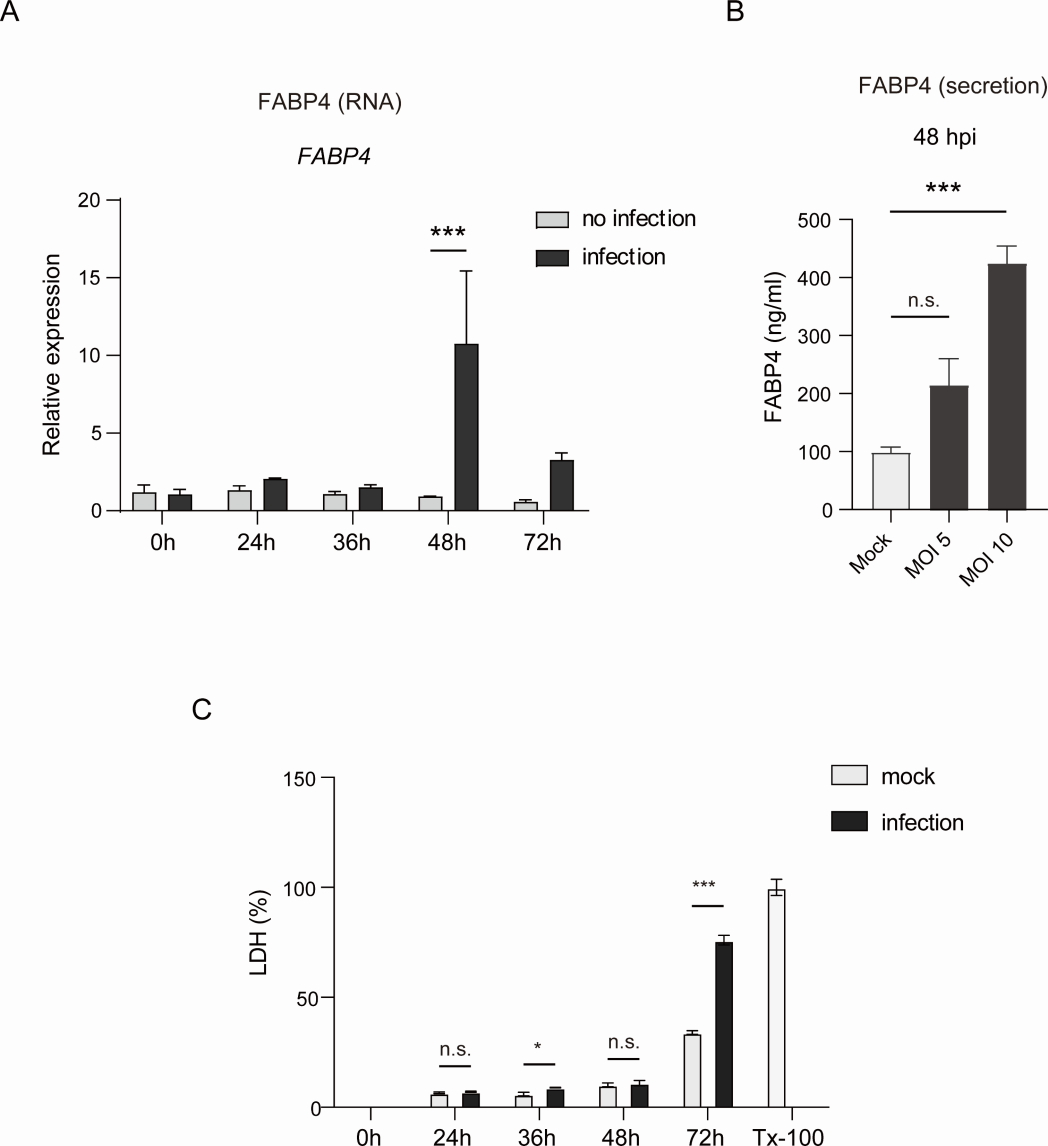
FABP4 regulation during *T. cruzi* infection in murine adipocytes. (**A**) Relative levels of *Fabp4* mRNA in 3T3-L1 adipocytes after mock or *T. cruzi* infection at the indicated hpi were determined by real-time PCR. *Gus* mRNA served as the internal control. (**B**) Secretion of FABP4 was measured in the cultured supernatants of 3T3-L1 adipocytes at 48 hpi of mock or *T. cruzi* (MOI of 5, MOI of 10) infection. A 4 h incubation with forskolin (20 µM) was used as the positive control for lipolysis. (**C**) A lactate dehydrogenase (LDH) assay was performed using the supernatant of 3T3-L1 adipocytes at 24, 36, and 48 hpi. Cells were incubated with 2% Triton-X-100 (Tx-100) for 2 hours to obtain maximum LDH release. A two-way ANOVA with Sidak’s multiple comparison test (**A and C**) and a one-way ANOVA with Tukey’s (**B**) were used to analyze multiple groups. *P < 0.05, **P < 0.01, ***P < 0.001, n.s., not significant.

The apparent dissociation between glycerol release and FFA release in *T. cruzi* infection-induced LD hydrolysis led us to examine whether lipid chaperone FABP4 plays any role in the intracellular trafficking of liberated FFAs and facilitates the growth of *T. cruzi* in adipocytes. To explore the role of intracellular FABP4 during *T. cruzi* infection, we first examined the kinetics of FABP4 protein expression (Fig. S1) and *Fabp4* mRNA expression (Fig. 2A) in 3T3-L1 adipocytes after *T. cruzi* infection. We found abundant FABP4 protein expression in the cell lysate of mature adipocytes during infection and an increase in *Fabp4* mRNA expression at 48 hpi. Immunoblot analysis of FABP4 in cultured supernatants (Sup) of 3T3-L1 adipocytes after *T. cruzi* infection revealed the rise of FABP4 in cultured supernatants at 48 hpi and 72 hpi (Fig. S1), which corroborates the previous reports showing FABP4 is actively secreted from adipocytes under fasting, lipolysis stimuli (21–23), and *C. pneumoniae* infection-induced lipolysis (24). We further confirmed that *T. cruzi* infection-induced secretion of FABP4 in the cultured medium of adipocytes was multiplicity of infection (MOI)-dependent (Fig. 2B). We excluded the possibility of FABP4 detection after host cell death by lactate dehydrogenase (LDH) cytotoxicity assay using culture supernatants of 3T3-L1 adipocytes after *T. cruzi* infection (Fig. 2C).

### FABP4 inhibition or genetic silencing decreases *T. cruzi* replication in adipocytes

**Fig 3:**
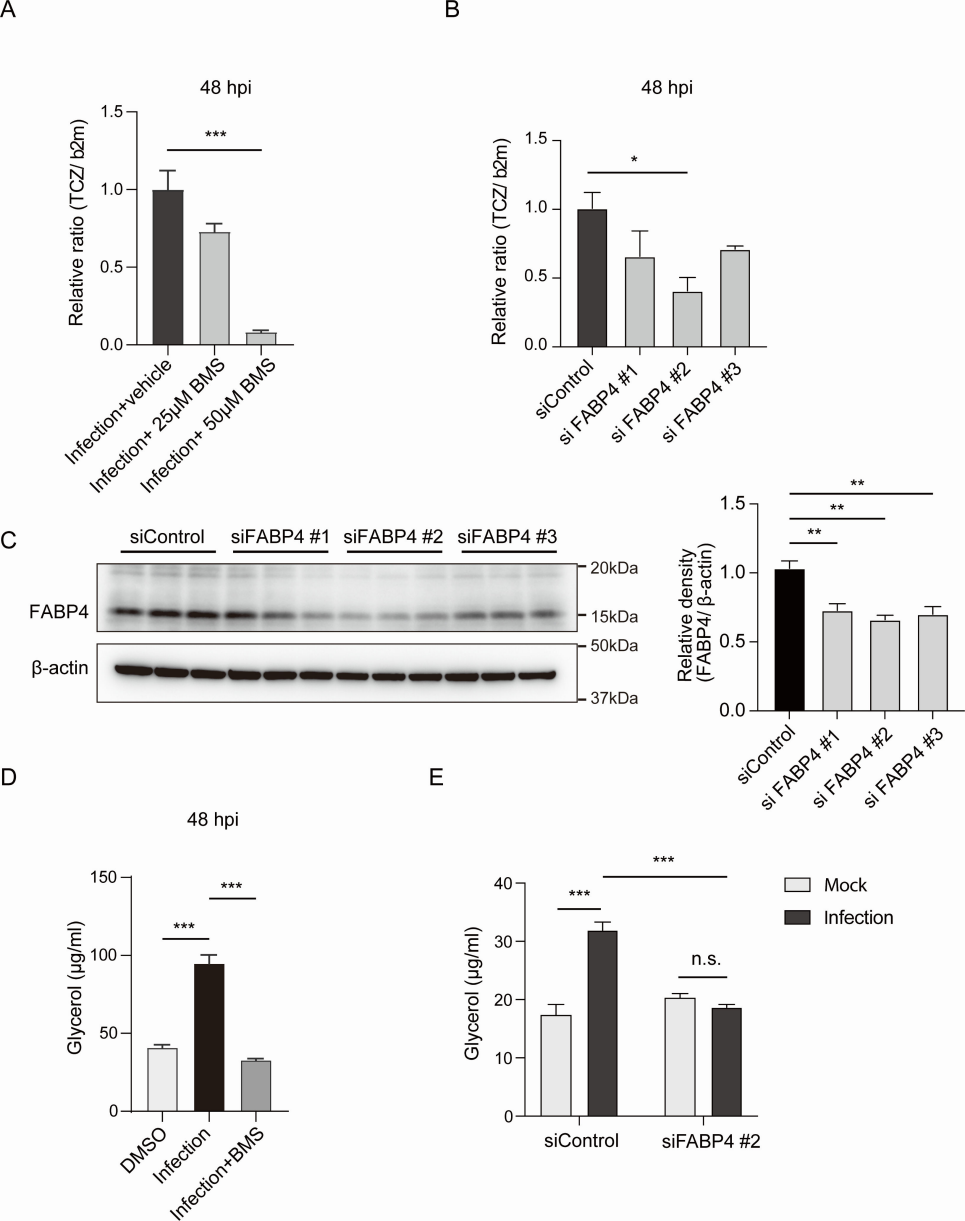
FABP4 inhibition or genetic silencing reduces *T. cruzi* replication in murine adipocytes. (**A**) The relative ratio of genomic DNA PCR of *T. cruzi* in 3T3-L1 adipocytes at 48 hpi in increasing doses of FABP4 chemical inhibitor BMS309403 (BMS) or vehicle control. (**B**) The relative ratio of *T. cruzi* genomic DNA PCR in 3T3-L1 adipocytes expressing siFABP4 or siControl at 48 hpi. (**C**) Western blot of FABP4 (15 kDa) and β-actin (42 kDa) protein levels in adipocytes expressing si*Fabp4* (#1, #2, and #3) or siControl. (**D**) Secretion of glycerol was measured in the cultured medium of *T. cruz*i-infected 3T3-L1 adipocytes at 48 hpi (MOI of 10) in the presence or absence of BMS309403 (BMS). (**E**) Secretion of glycerol from siRABP4-treated or siControl-treated adipocytes at 48 hpi after mock or *T. cruzi* infection. The data are shown as mean ± SEM and are representative of at least three experiments. For the analysis of multiple groups, a one-way ANOVA with Tukey’s multiple comparison test (A, B, C, and D) and a two-way ANOVA with Sidak’s multiple comparison test (**E**) were used. *P < 0.05, **P < 0.01, ***P < 0.001, n.s., not significant.

The apparent dissociation between glycerol release and FFA release in *T. cruzi* infection-induced LD hydrolysis (Fig. 1D and E) led us to examine whether lipid chaperone FABP4, which is abundantly expressed in adipocytes, plays a role in intracellular trafficking of liberated FFAs and facilitates the growth of *T. cruzi* in adipocytes. To understand the functional role of FABP4 in *T. cruzi* infection in adipocytes, we used a small molecule inhibitor against FABP4: BMS309403 (25, 26). BMS309403 has been reported to bind to FABP4 with a K_p_ of 552 nM (26) and has been shown to block FABP4’s ability to bind its lipid ligand (17). We found the treatment with FABP4 inhibitor (BMS309403) abrogates the intracellular growth of *T. cruzi* in murine adipocytes (Fig. 3A). Genetic silencing of *Fabp4* by siRNA also reduced the growth of *T. cruzi* in adipocytes (Fig. 3B). We validated the efficiency of gene silencing of *Fabp4* in these adipocytes by western blot analysis (Fig. 3C). It is of note that FABP4 inhibition or genetic deletion reduces glycerol release from infected adipocytes, indicating that FABP4 also plays a role in LD hydrolysis (lipolysis) as reported previously (27, 28), in addition to the role in intracellular trafficking of liberated FFAs (Fig. 3D and E).

### FABP4-dependent host mitochondrial fatty acid oxidation is essential for *T. cruzi* proliferation in murine adipocytes

**Fig 4:**
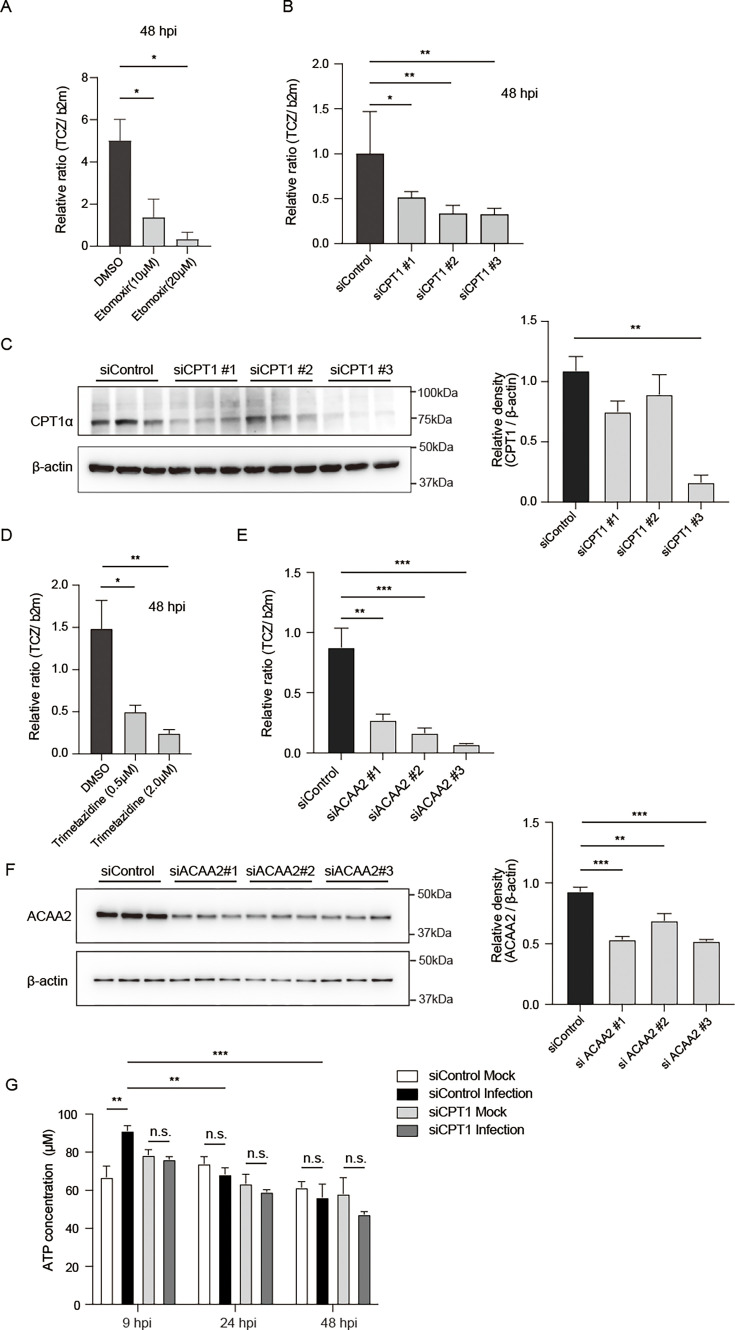
FABP4-dependent FAO is necessary for the proliferation of *T. cruzi* in adipocytes. (**A**) Relative ratio of *T. cruzi* DNA PCR in 3T3-L1 adipocytes at 48 hpi after *T. cruzi* infection in the presence of Etomoxir (10, 20 µM) or DMSO control. (**B**) The relative ratio of *T. cruzi* DNA PCR in 3T3-L1 adipocytes expressing si*Cpt1a* (#1~#3) or siControl at 48 hpi. (**C**) Western blot of CPT1A (~78 kDa) and β-actin protein (42 kDa) levels in adipocytes expressing si*Cpt1a* (#1, #2, and #3) or siControl (left), and the statistical analyses (right). (**D**) The relative ratio of *T. cruzi* DNA PCR in 3T3-L1 adipocytes at 48 hpi in the presence of Trimetazidine (0.5, 2.0 µM) or dimethyl sulfoxide (DMSO) control. (**E**) The relative ratio of *T. cruzi* DNA PCR in 3T3-L1 adipocytes expressing si*ACAA2*(#1~#3) or siControl at 48 hpi. (**F**) Western blot of ACAA2 (42 kDa) and β-actin protein levels in adipocytes expressing si*ACAA2* (#1, #2, and #3) or siControl (left), and the statistical analyses (right). (**G**) Cytosolic ATP levels of 3T3-L1 adipocytes expressing si*Cpt1a* or siControl at 9 hpi, 24 hpi, and 48 hpi after *T. cruzi* (MOI of 10) or mock infection. The data are shown as mean ± SEM and are representative of at least three experiments. A one-way ANOVA with Tukey’s multiple comparison test (A, B, C, D, E, and F) and a two-way ANOVA with Tukey’s multiple comparison test (**G**) were used to analyze multiple groups. *P < 0.05, **P < 0.01, ***P < 0.001, n.s., not significant.

To further understand how FABP4 facilitates the proliferation of *T. cruzi* in adipocytes, we examined the possibility that FABP4 transports liberated FFA to host mitochondria for fatty acid oxidation (FAO). We found that treatment with Etomoxir, which inhibits carnitine palmitoyltransferase 1A (CPT1A), the rate-limiting enzyme for FAO, significantly inhibits the intracellular proliferation of *T. cruzi* amastigote in adipocytes (Fig. 4A). To rule out the possibility that Etomoxir inhibits the CPT1A homolog in *T. cruzi* (29, 30), we investigated the effect of Etomoxir on the proliferation of *T. cruzi* epimastigote forms. Concentrations of 10 µM or 20 µM Etomoxir, which significantly reduced the intracellular growth of *T. cruzi* amastigote in adipocytes, did not affect the proliferation of *T. cruzi* epimastigote forms in LIT medium culture (Fig. S2A and C), although a very high dose of Etomoxir (500 µM) impaired epimastigote proliferation as reported by Souza et al. (31). We also generated 3T3-L1 adipocytes expressing siRNA Cpt1a or control siRNA to reinforce the inhibitory effect of Etomoxir on the intracellular proliferation of *T. cruzi* amastigote in adipocytes. We observed that *T. cruzi* amastigote proliferation in 3T3-L1 adipocytes was significantly reduced by the genetic knockdown of host Cpt1a (Fig. 4B), although the efficiency of siRNA-mediated knockdown varies among the three different siRNA sequences, as shown by target protein expression levels (Fig. 4C).

Trimetazidine is a selective inhibitor of mitochondrial long-chain 3-ketoacyl-CoA thiolase, the final enzyme in the FAO pathway, also known as mitochondrial acetyl-Coenzyme A acyltransferase 2 (ACAA2). We found that pharmacological inhibition of FAO by Trimetazidine prevents the intracellular growth of *T. cruzi* in murine adipocytes (Fig. 4D), while the same concentration of Trimetazidine did not inhibit the proliferation of *T. cruzi* epimastigote forms (Fig. S2B and C). To further confirm that host cell FAO is essential for the optimal intracellular growth of *T. cruzi* amastigotes in adipocytes, we prepared 3T3-L1 adipocytes expressing siRNA for ACAA2 or a control siRNA. We found that *T. cruzi* proliferation in 3T3-L1 adipocytes was significantly reduced by knocking down host ACAA2 (Fig. 4E). The effectiveness of siRNA silencing was confirmed by the decrease in protein levels (Fig. 4F). These results indicate that infection-induced FAO in host cells is crucial for the intracellular proliferation of *T. cruzi* amastigotes in adipocytes.

In line with these observations, cytosolic ATP levels in 3T3-L1 adipocytes increased at 9 hpi, which was prevented by genetic silencing of Cpt1a (Fig. 4G). Notably, *T. cruzi* infection did not lead to an increase in cytosolic ATP levels at 24 hpi or 48 hpi. It is well known that a proper mitochondrial membrane potential is essential for efficient ATP production and mitochondrial health. We used flow cytometry staining to determine mitochondrial function based on mitochondrial mass (MitoTracker Green) and mitochondrial membrane potential (MitoTracker Red). We observed that the proportion of cells stained with MitoGreen^high^ and MitoRed^low^, representing the percentage of depolarized, dysfunctional mitochondria-containing cells, increased at 24 hpi and 48 hpi compared to mock infection (Fig. S3A). These results suggest that the lack of cytosolic ATP increase at these time points is due to a decrease in host mitochondrial membrane potential in adipocytes. In addition, we found that host mitochondrial ROS levels increased at 24 hpi and 48 hpi (Fig. S3B), indicating that excessive ROS disrupts mitochondrial integrity and impairs ATP synthesis (32, 33). Although we do not exclude the other possibility in which liberated FFA might be transported and directly up-taken by parasites (34, 35), our current results suggest that liberated FFAs, if not all, are rendered for host mitochondrial FAO via FABP4-mediated intracellular lipid trafficking and that *T. cruzi* usurps this FABP4-dependent host mitochondrial FAO for the intracellular proliferation of parasite amastigotes.

### FABP4-dependent host mitochondrial FAO increases oxidative stress and promotes *T. cruzi* proliferation in mouse adipocytes

**Fig 5:**
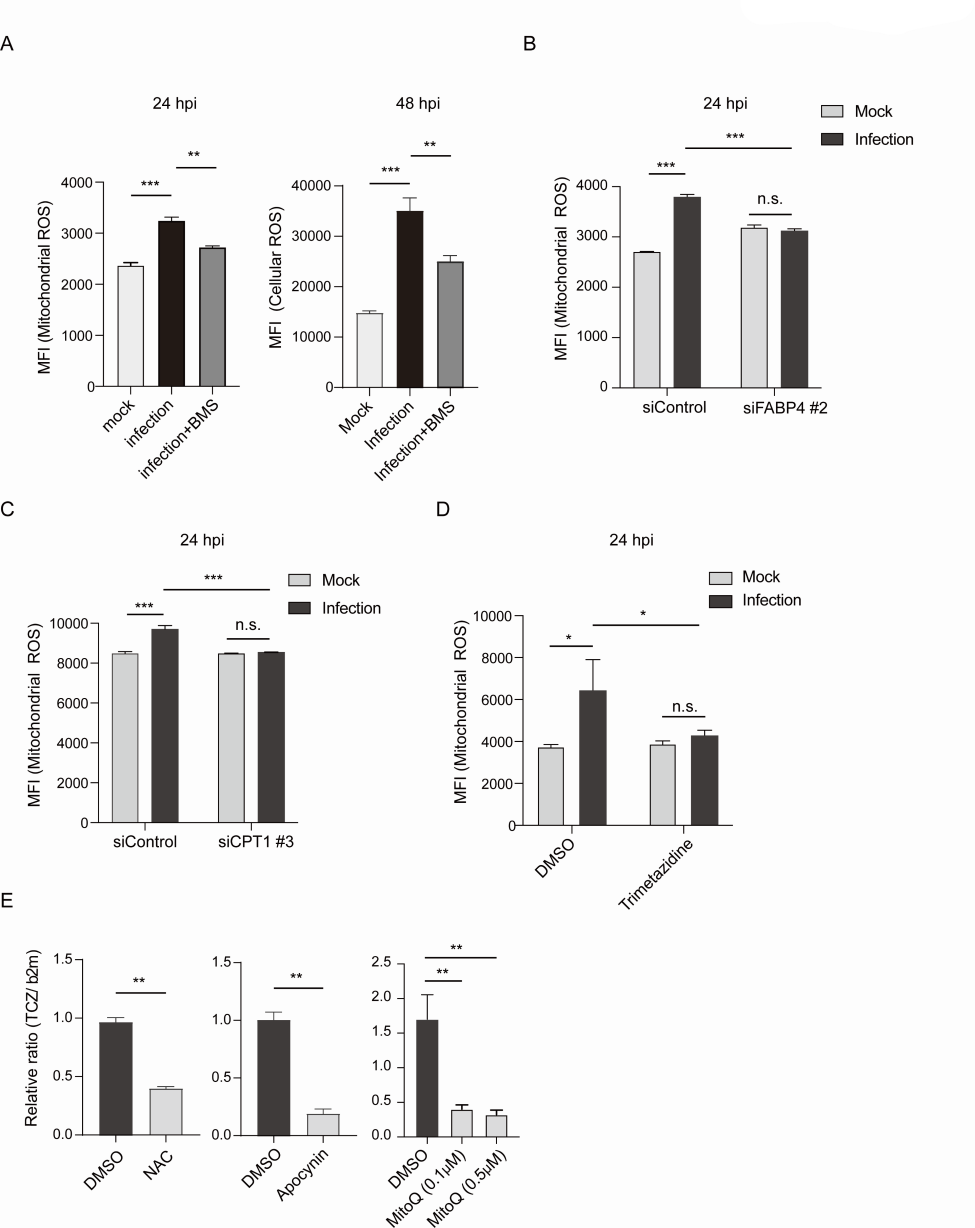
FABP4-dependent FAO increases oxidative stress and promotes *T. cruzi* proliferation in murine adipocytes. (**A**) The effect of FABP4 chemical inhibition by BMS309403 (BMS) on mitochondrial ROS (at 24 hpi) and cellular ROS (at 48 hpi) of 3T3-L1 adipocytes after mock or *T. cruzi* infection (MOI of 10) was determined by flow cytometric analysis of mitoSOX and CellROX, respectively. (**B and C**) Effects of genetic silencing of FABP4 (**B**) or CPT-1 (**C**) on mitochondrial ROS in adipocytes at 24 hpi after mock or *T. cruzi* infection. (**D**) Effect of pharmacological inhibition of FAO by Trimetazidine on mitochondrial ROS of 3T3-L1 adipocytes at 24 hpi after mock or *T. cruzi* infection. (**E**) Effect of ROS scavenger: N-acetyl cysteine (NAC), NADPH oxidase inhibitor: Apocynin, or mitochondrial ROS scavenger: MitoQ, on *T. cruzi* genomic DNA PCR of 3T3-L1 adipocytes at 48 hpi after *T. cruzi* infection. The relative ratio to the dimethyl sulfoxide (DMSO) solvent control is shown. The data are shown as mean ± SEM and are representative of at least three experiments. For the analysis of multiple groups, a one-way ANOVA with Tukey’s multiple comparison test (A and E; right panel) and a two-way ANOVA with Sidak’s multiple comparison test (B, C, and D) were used. An unpaired *t*-test (E; left and middle panel) was used to analyze the two groups. *P < 0.05, **P < 0.01, ***P < 0.001, n.s., not significant

Next, we examined how the increase in FABP4-dependent FAO leads to the intracellular proliferation of *T. cruzi* amastigotes in adipocytes. The host mitochondrial FAO produces ATP. However, *T. cruzi* amastigotes do not directly uptake ATP from host cells, but instead rely on the uptake of nucleosides and nucleobases for their purine and pyrimidine requirements (9, 36). It has been reported that *T. cruzi* utilizes reactive oxygen species (ROS) to proliferate in host cells (10, 12, 37). It is well known that mitochondria are the primary cellular source for generating ROS, and most ROS are produced in the electron transport chain present in the inner mitochondrial membrane via oxidative phosphorylation, producing ATP (38). However, it has not yet been verified whether ROS enhances the proliferation of *T. cruzi* amastigotes in adipocytes. Therefore, we examined the host mitochondrial ROS and cellular ROS levels in adipocytes after *T. cruzi* infection. We observed that mitochondrial ROS and cellular ROS levels increase in adipocytes after *T. cruzi* infection (Fig. S3B), which are significantly reduced by chemical inhibition of FABP4 (Fig. 5A). We confirmed that genetic silencing of *Fabp4* or *Cpt1a* abrogates *T. cruzi* infection-induced elevation of mitochondrial ROS (Fig. 5B and C). Chemical inhibition of FAO by Trimetazidine also abolished the elevation of mitochondrial ROS after infection (Fig. 5D). Notably, the treatment with ROS scavenger N-acetyl cysteine (NAC), NADPH oxidase inhibitor Apocynin, or mitochondrial ROS scavenger MitoQ decreased the intracellular growth of *T. cruzi* in adipocytes (Fig. 5E). These results suggest that FABP4-dependent FAO fuels respiratory stress, and the increase of ROS contributes to the growth of *T. cruzi* amastigotes in adipocytes.

### Cellular iron is mobilized by oxidative stress induced by FABP4-dependent host FAO, which facilitates *T. cruzi* infection in mouse adipocytes

**Fig 6:**
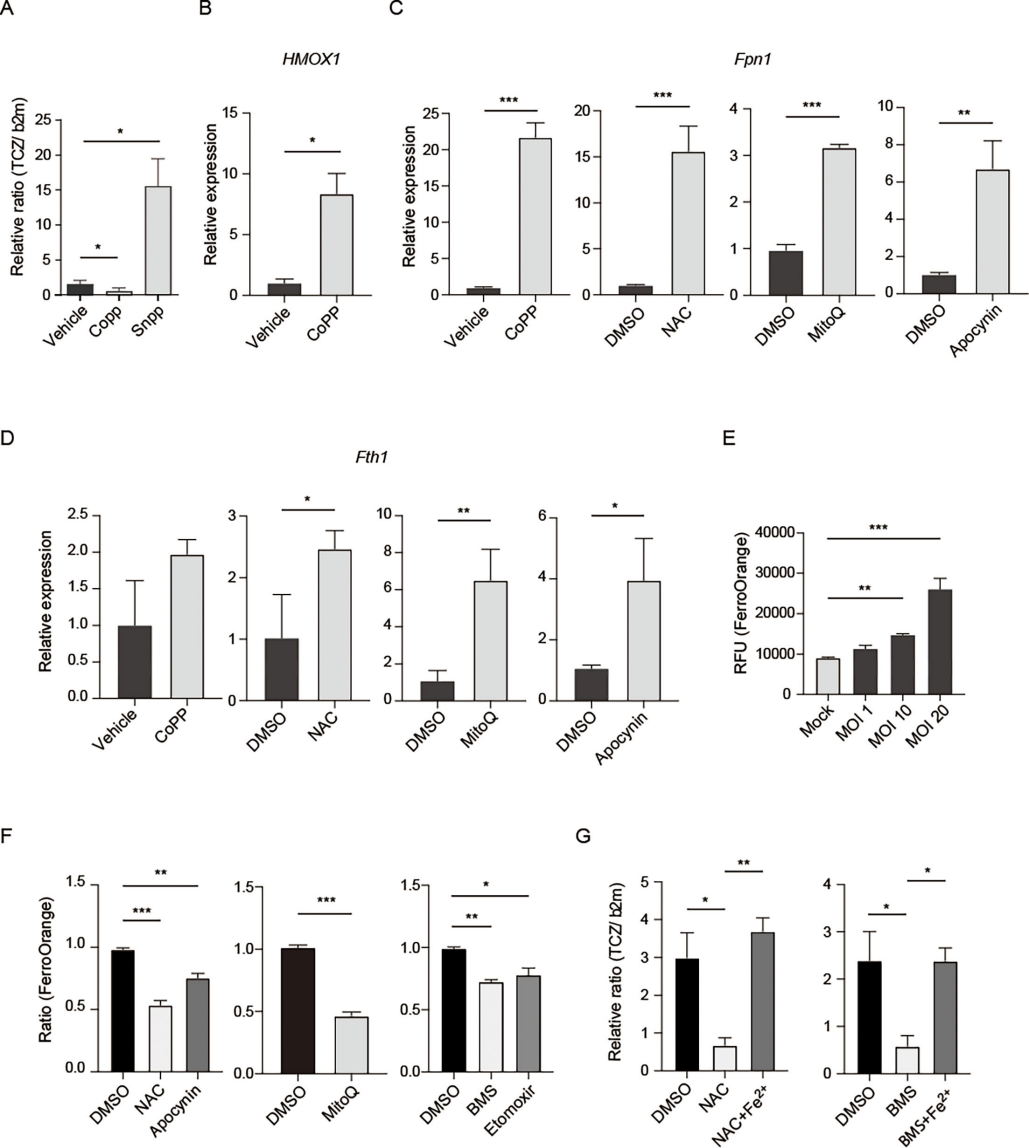
Cellular iron is mobilized by oxidative stress induced by FABP4-dependent FAO, which fuels *T. cruzi* infection in murine adipocytes. (**A**) Effects of the NRF2/HO-1 inducer, CoPP, and the HO-1 inhibitor, SnPP, on the intracellular growth of *T. cruzi* in 3T3-L1 adipocytes at 48 hpi were determined by quantitative genomic DNA PCR of *T. cruzi*. (**B**) Effect of treatment with antioxidants: CoPP on the mRNA expression for HO-1 (*Hmox1*) in 3T3-L1 adipocytes at 48 hpi after infection with *T. cruzi*. (**C, D**) Effects of CoPP, NAC, MitoQ, or Apocyanin on the expression levels of mRNA for Ferroportin (*Fpn1*) (**C**) and Ferritin (*Fth1*) (**D**) in 3T3-L1 adipocytes at 48 hpi. (**E**) Cytosolic Fe^2+^ levels in 3T3-L1 adipocytes at 24 hpi after *T. cruzi* infection with an increasing number of MOI were determined by FerroOrange MFI. (**F**) Effect of NAC, Apocynin, MitoQ, BMS309403 (BMS), and Etomoxir on FerroOrange MFI levels (cellular Fe^2+^) in adipocytes at 24 h after *T. cruzi* infection (MOI 10). The relative ratio of FerroOrange MFI to solvent dimethyl sulfoxide (DMSO) control was shown. (**G**) Effect of the treatment with Ferrous sulfate on anti-oxidant NAC (left panel) and BMS309403- (right panel) induced inhibition of *T. cruzi* growth in adipocytes was determined by quantitative genomic DNA PCR of *T. cruzi*. The data are shown as mean ± SEM and represent at least three experiments. A one-way ANOVA with Tukey’s multiple comparison tests (A, E, F; left panel, and G) was used to analyze multiple groups. An unpaired t-test (B, C, D, and F; right panel) was used to analyze two groups. *P < 0.05, **P < 0.01, ***P < 0.001, n.s., not significant.

*T. cruzi* relies on various metabolic strategies for energy transduction. Iron is vital for the parasite’s energy metabolism because it participates in its mitochondrial function and ATP formation (39). Paiva et al. reported that cellular iron is mobilized by oxidative stress and fuels *T. cruzi* infection in macrophages (12). Nuclear factor erythroid-derived 2-like 2 (NRF2) coordinates antioxidant defenses, including heme-oxygenase-1 (HO-1) expression. Ferritin and ferroportin contain antioxidant response element motifs in their promoters and are increased by NRF2 activators. Ferritin-bound iron is a redox-inert storage form of iron. Paiva et al. showed that cobalt protoporphyrin (CoPP), an NRF2/HO-1 inducer, decreases macrophage parasitism (12). Therefore, we examined the effects of the NRF2/HO-1 inducer CoPP on *T. cruzi* growth in murine adipocytes. Similar to macrophages reported by Paive, we found that CoPP reduces *T. cruzi* growth in adipocytes, while the HO-1 inhibitor tin protoporphyrin (SnPP) increases *T. cruzi* growth in adipocytes (Fig. 6A). CoPP decreased *T. cruzi* growth in adipocytes by activating NRF2 target genes (*Hmox1*) (Fig. 6B). Furthermore, treatment with antioxidants (CoPP/NAC/Apocynin) in *T. cruzi*-infected 3T3-L1 adipocytes increased the mRNA levels of Ferroportin (*Fpn1*) and Ferritin (*Fth1*), which are an iron exporter and the protein responsible for cytosolic iron storage, respectively (Fig. 6C and D). To further confirm that cellular iron is mobilized by oxidative stress and fuels *T. cruzi* infection in adipocytes, we measured the intracellular Fe^2+^ labile iron pool in 3T3-L1 adipocytes during infection. FerroOrange MFI levels, indicating intracellular ferrous ion (Fe^2+^) levels, increased after *T. cruzi* infection in an MOI-dependent manner (Fig. 6E). Importantly, treatment with anti-oxidants (NAC, Apocynin, or MitoQ) prevented the increase of intracellular labile iron (Fe^2+^) in adipocytes after *T. cruzi* infection (Fig. 6F), which aligns with the anti-oxidants' inhibitory effect on the intracellular growth of *T. cruzi* in adipocytes (Fig. 5E). Lastly, incubation with Fe^2+^ reversed the antioxidant NAC-mediated decrease in parasite growth, as well as the parasite growth inhibition caused by chemical inhibition of FABP4 (Fig. 6G). These results suggest that *T. cruzi* infection-induced increase in cellular ROS, which FABP4-dependent FAO promotes, raises the labile iron pool in 3T3-L1 adipocytes, leading to *T. cruzi* proliferation.

## DISCUSSION

**Fig 7:**
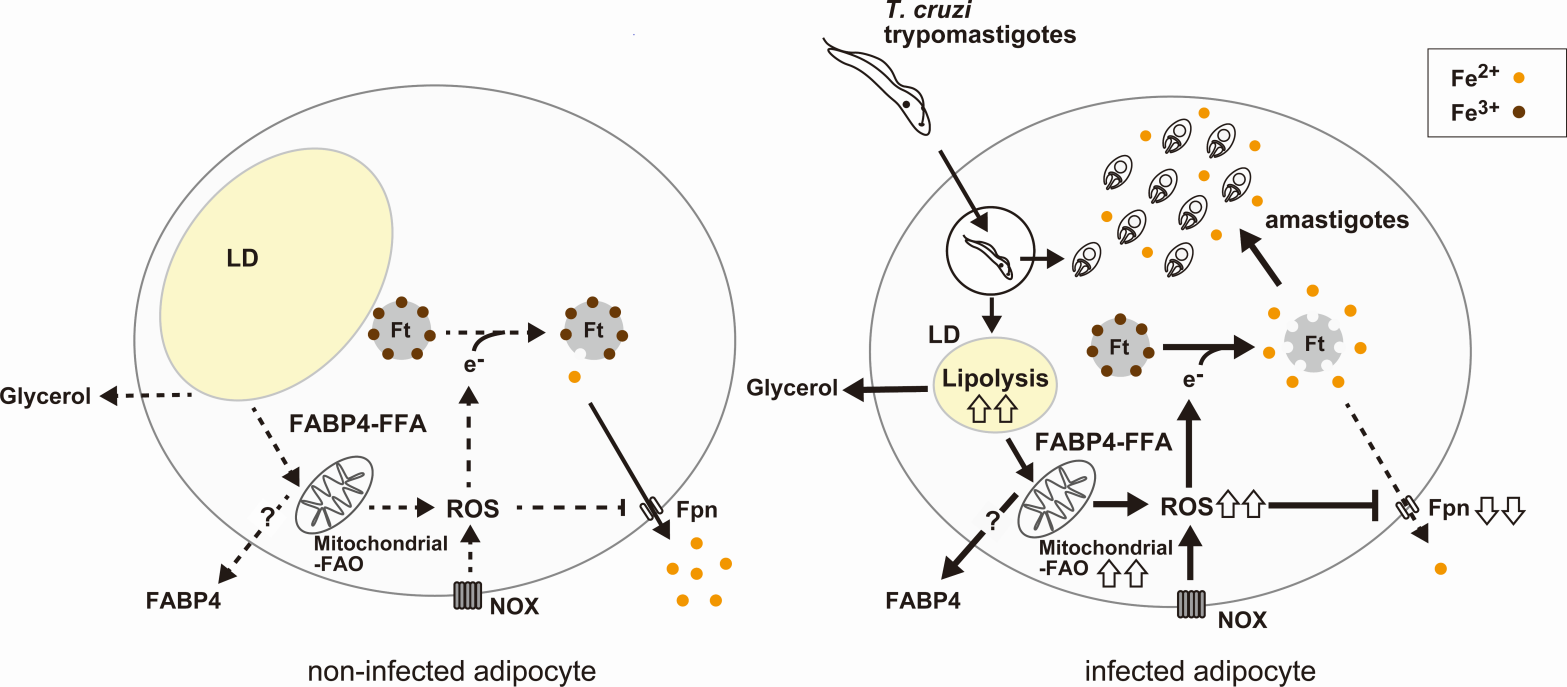
Schematic of FABP4 promoting FAO-mediated oxidative stress to mobilize cellular iron and promote parasite growth in murine adipocytes. In the cytosol, iron is stored as ferric iron (Fe^3+^) associated with ferritin (Ft) or can be exported from the cell as ferrous iron (Fe^2+^) through ferroportin (Fpn), an iron exporter. The expression of ferroportin and ferritin increases with antioxidants, which can reduce the levels of the labile iron pool (Fe^2+^) in the cytosol. Triacylglycerol in lipid droplets (LD) in adipocytes is degraded upon infection with *T. cruzi*. The released free fatty acids (FFAs) are transported to mitochondria in an FABP4-dependent way, and the subsequent mitochondrial FAO raises host mitochondrial and cellular ROS. A ferrous form of iron (Fe^2+^) is released from ferritin in environments with high cellular ROS, and this boosted labile iron pool is taken up by *T. cruzi* amastigotes, helping the parasite grow. Ft, ferritin; Fpn, ferroportin; NOX, NADPH oxidase; ROS, reactive oxygen species; LD, lipid droplet; FFA, free fatty acid; FAO, fatty acid oxidation.

In the present study, we demonstrated that *T. cruzi* amastigotes’ proliferation in murine adipocytes correlates with the degradation of lipid droplets of adipocytes. We found that liberated FFA was FABP4-dependently transported to the host cell’s mitochondria and used for FAO. The pivotal roles of FABP4 and FAO in *T. cruzi* infection in adipocytes were confirmed by genetic silencing of FABP4 or CPT-1 and using pharmacological inhibition of FABP4 or CPT-1. This increased FABP4-dependent FAO of the host cell’s mitochondria upon *T. cruzi* infection leads to mitochondrial ROS generation in infected cells, mobilizing cellular iron and promoting parasite growth in adipocytes, as depicted in Fig. 7.

In *T. cruzi* infection-induced lipolysis, there was an apparent dissociation between the release of glycerol and FFAs, indicating that liberated FFAs are utilized intracellularly for the growth of parasites. We demonstrated that FABP4-mediated transport of liberated FFAs to host mitochondria and subsequent fatty acid β-oxidation is required for the proliferation of *T. cruzi* in murine adipocytes. Cellular ATP levels increased in adipocytes at 9 h after infection with *T. cruzi*. However, *T. cruzi* can produce ATP by itself and does not need to obtain ATP from host cells, while *C. pneumoniae* cannot make ATP by itself and requires ATP from host cells, as we previously reported (15). Therefore, the requirement of FAO for *T. cruzi* to proliferate in adipocytes suggests there must be some other mechanism of FAO-induced proliferation of *T. cruzi*. We found that fatty acid-fueled mitochondrial ROS production induces the mobilization of cellular iron, facilitating parasitism in adipocytes. The current result extends the previous report of *T. cruzi*-infected macrophages (12). It can be safely said that the stimulation of *T. cruzi* growth by oxidative stress would not be restricted to macrophages but also to other cell types, such as adipocytes and probably cardiomyocytes (40), in which a persistent oxidative environment can be generated by persistent infection by the parasites.

Despite the elevations of cellular ROS and the labile iron pools in 3T3-L1 adipocytes after infection with *T. cruzi*, infected cells at 48 hpi were strikingly resistant to cell death (Fig. 2C). Cells at 48 hpi were packed with amastigote nests (Fig. 1A). Why and how did these infected adipocytes escape ferroptotic cell death under oxidative stress and increased labile iron (41)? It has been known that *T. cruzi* has several enzymes destined to deactivate reactive oxygen species, namely, peroxidases, iron superoxide dismutases, and hydroperoxidases (42). These enzymes allow the parasite’s survival and protect the host cell from ROS and nitrogen species. Alternatively, some of the ATP generated by host β-oxidation may help promote host cell survival to give the time to develop, whereas premature host cell mortality would prevent *T. cruzi* expansion (43).

Several other mechanisms explain the discrepancy between glycerol and FFA release after *T. cruz*i infection-induced LD hydrolysis. FFAs can be transported to and taken up by *T. cruzi* amastigotes (34, 35, 44). Gazos-Lopes et al. elegantly showed that lipidome analysis of intracellular *T. cruzi* and their mammalian host cells reveals that the FAs signatures in the triacylglycerol pools are very similar between them, indicating parasites may acquire fatty acids from host triacylglycerol by some unknown mechanism (45). Further studies, such as the trafficking of fatty acids in *T. cruzi*-infected adipocytes using BODIPY Fl C 12, will be required to address these questions. Alternatively, some portion of liberated FFAs can be re-esterified and utilized to form micro-lipid droplets, which can be further degraded by lipolysis (46). Gazos-Lopes et al. reported reduced *T. cruzi* amastigote proliferation in diacylglycerol acyltransferases (DGAT1/2)-deficient fibroblasts, underscoring the importance of host triacylglycerol pools for parasite intracellular growth (45). We also observed that a chemical inhibitor of DGAT-1 decreased *T. cruzi* proliferation in adipocytes (data not shown), supporting this possible pathway.

This study demonstrated that *T. cruzi* infection in adipocytes induces FABP4 secretion associated with lipolysis. It has been reported that the release of FABP4 from adipocytes may be involved in the development of cardiac contractile dysfunction in obese subjects (47). More recently, FABP4 has been demonstrated to serve as a secreted hormone capable of interacting with other proteins, such as adenosine kinase (ADK) and nucleoside diphosphate kinase (NDPK), and to establish direct endocrine links between adipose tissue and distant organs (48). Although we have not clarified the mechanism of FABP4 secretion from *T. cruzi*-infected adipocytes, it is reasonable to speculate that secreted FABP4 from *T. cruzi*-infected adipocytes, which is known to harbor the parasites for extended periods, may play a role in the pathogenesis of Chagas cardiomyopathy associated with chronic *T. cruzi* infection. Thus, our findings provide a foundation for future studies to examine the *in vivo* significance of FABP4 secretion on the pathogenesis of Chagas cardiomyopathy caused by persistent *T. cruzi* infection.

In this work, we focused on the role of liberated FFAs on host mitochondrial FAO and the subsequent oxidative stress, which is followed by iron mobilization and *T. cruzi* proliferation in adipocytes. We have not examined the possible mechanisms of lipid droplet (LD) breakdown (lipolysis) in adipocytes after infection with *T. cruzi*. Nagajyothi et al. found the upregulation of key lipolytic enzymes such as adipose triglyceride lipase (ATGL), hormone-sensitive lipase (HSL), and lipoprotein lipase (LPL) in both brown and white adipose tissue during acute infection (18), while González et al. reported that the expression of the main lipolytic enzymes was sharply downregulated *in vivo* and *in vitro* 3T3-L1 adipocytes (19). In addition to lipolytic degradation of LD by cytosolic lipases, the LD degradation pathway by LD-associated perilipin (PLIN2 and PLIN3) chaperone-mediated autophagy degradation (CMA) or microlipophagy has also been reported (49, 50). We are investigating the relative importance of these LD breakdown pathways and the regulation of LD biogenesis and turnover in 3T3-L1 adipocytes during *T. cruzi* infection.

Metabolomic evidence supporting FAO-ROS reliance in adipocyte-dwelling *T. cruzi* comes from studies demonstrating the parasite’s ability to utilize fatty acids as a primary energy source, especially under nutrient-limited conditions, and the connection between FAO and mitochondrial ROS production (36). A. M. Silber’s group showed that *T. cruzi* epimastigotes switch from glucose consumption to FAO when glucose is scarce (31). In their *in vitro* culture model of epimastigotes, they demonstrated that blocking CPT1 with etomoxir significantly reduces O_2_ consumption, mitochondrial activity, and ATP levels in the parasite, emphasizing *T. cruzi*’s reliance on FAO for energy balance. Although understanding of amastigote metabolism is still limited, these results are highly relevant to intracellular amastigotes in adipocytes, where similar nutrient shortages and metabolic challenges are present. Future research into the biology and metabolism of amastigotes may offer valuable insights for developing stage-specific drugs targeting clinically important stages of clinical relevance.

In summary, our study reveals the pivotal role of FABP4 in *T. cruzi* replication within adipocytes. This is achieved through its involvement in lipolysis and the transportation of liberated free fatty acids to the host cell’s mitochondria, where they are utilized for FAO. The resulting infection-induced mitochondrial FAO fuels ROS, leading to accelerated parasite replication. These findings not only deepen our understanding of the *T. cruzi* infection process but also hold promise for the development of novel therapeutic strategies. By targeting FABP4, we may be able to effectively combat *T. cruzi* infection. Our ongoing *in vivo* studies using *FABP4*^−/−^ mice will further validate the significance of our findings in a living organism.

## MATERIALS AND METHODS

### Reagents and antibodies

Reagents: Forskolin (Sigma Aldrich, St Louis, MO), BMS309403 (Selleck Chemicals, Houston, TX), Etomoxir (Sigma Aldrich), 1-(2,3,4-Trimethoxybenzyl)-piperazine dihydrochloride (Trimetazidine, TCI, Japan), N-Acetyl-L-cysteine (NAC, FUJIFILM Wako, Japan), Apocynin (Abcam, UK), Co (III) Protoporphyrin IX, Chloride (CoPP, Frontier Specialty Chemicals Inc., Logan, UT)**,** Sn (IV) Protoporphyrin IX Dichloride (SnPP, Frontier Specialty Chemicals Inc.), Mitoquinone mesylate (MitoQ, MedChemExpress, Monmouth Junction, NJ), and FeSO_4_ (Sigma Aldrich).

Antibodies: Polyclonal goat antibody against FABP4 (R&D Systems Inc., Minneapolis, MN), monoclonal mouse antibody against β-actin (Santa Cruz Biotechnology, Dallas, TX), monoclonal mouse antibody against CPT1A (Abcam), and rabbit monoclonal antibody against ACAA2 (Abcam).

### Parasites

*T. cruzi* Tulahuen strain trypomastigotes were kindly provided by Professor Shinjiro Hamano (Nagasaki University, Japan) (51) and were maintained in tissue culture of LLC-MK2 cells (ATCC, CCL-7). *T. cruzi* trypomastigotes released from the infected host cells were collected from the supernatant of the infected cells on the fifth day after infection and used for experiments or to continue parasite culture. The epimastigote of *T. cruzi* was maintained in the logarithmic growth phase by continuous culture for 72 hours in Liver Infusion Tryptose (LIT) medium at 28°C (52).

### *In vitro T. cruzi* infection in adipocytes and determination of parasite burden

3T3-L1 differentiated adipocytes (5 × 10^4^) were infected with *T. cruzi* at a multiplicity of infection (MOI) of 10 and used for experiments at 48 hpi, except as explicitly indicated in the figure. The following drugs were added at the same time as the infection. BMS309403 (BMS; 25 µM, 50 µM), Etomoxir (10 µM, 20 µM), Trimetazidine (0.5 µM, 2.0 µM), NAC (5 mM), Apocynin (100 µM), MitoQ (0.1 µM, 0.5 µM) were dissolved in dimethyl sulfoxide (DMSO). CoPP (50 µM) and SnPP (50 µM) were dissolved in 0.2M NaOH and neutralized to pH 7.2 with 1M HCl. FeSO_4_ (100 µM) was dissolved in water. To determine parasite burdens in *T. cruzi*-infected adipocytes, DNA was purified from infected cells using ISOGENOME (NIPPON GENE, Japan) and parasite levels were determined by PCR (QuantStudio 3, Applied Biosystems Inc., Waltham, MA) using *T. cruzi* 195 bp repeat DNA-specific primers TCZ-F: 5′-GCT CTT GCC CAC AAG GGT GC-3′ and TCZ-R: 5′-CCA AGC AGC GGA TAG TTC AGG-3′ (53). According to the manufacturer’s instructions, PCRs were performed using THUNDERBIRD SYBR qPCR Mix (TOYOBO, Japan). At the same time, levels of the mouse β_2_-microglobulin gene were measured using primers β_2_m-F: 5′-TGG GAA GCC GAA CAT ACT G-3′ and β_2_m -R: 5′-GCA GGC GTA TGT ATC as internal controls for DNA input.

### *Proliferation assay* of *T. cruzi* epimastigote forms

Exponentially growing *T. cruzi* epimastigotes (1 × 10^6^ mL^−1^) were treated with BMS309403, Etomoxir, Trimetazidine, MitoQ, Apocynin, and NAC in LIT medium. Parasites were transferred to 96-well plates and incubated at 28°C. Cell growth was microscopically directly counted or measured by optical density (OD) at 595 nm every 24 hours for 7 days and quantified. The OD values were converted to cell counts using a linear regression equation previously obtained under the same conditions (31).

### 3T3-L1 adipocyte culture, differentiation, and Density-based separation, followed by Re-plating of Enriched Adipocytes in a Monolayer (DREAM)

3T3-L1 cells (ATCC, CL-173) were cultured in Dulbecco’s modified Eagle medium (DMEM) supplemented with 10% fetal bovine serum (Gibco, Thermo Fisher Scientific, Inc., Waltham, MA). 3T3-L1 cells were seeded in a 100 mm dish to induce differentiation into adipocytes and cultured for 2 days after reaching confluence. AdipoInducer reagent (Takara Bio, Japan) was added to DMEM- 10% FBS to induce adipocyte differentiation. From day 8 to day 10, differentiated 3T3-L1 cells were re-seeded to enrich the mature adipocytes as previously described (15, 54). The cells were washed once with phosphate-buffered saline (PBS), treated with 0.05% trypsin-EDTA (PBS) at 37°C for 5 minutes, and left until most cells had detached from the culture dish. The detached cells were centrifuged at 400 × *g* for 5 minutes and then resuspended in a medium consisting of DMEM containing 10% FBS and Histopaque-1077 (Sigma Aldrich) in a 1:1 ratio. The cell suspension was filtered through a 100 µm cell strainer (BD Falcon, Franklin Lakes, NJ) and centrifuged at 400 × *g* for 10 minutes. The floating cells containing the differentiated adipocytes were collected into a new tube, washed once with DMEM containing 10% FCS, and reseeded in a 24-well plate (5 × 10^4^ cells/well) for transfection.

### Introduction of siRNA into adipocytes

Adipocytes were transiently knocked down for CPT1a, FABP4, or mitochondrial acetyl-CoA acyltransferase (ACAA2) using mouse CPT1a siRNA (#1: S64345, Ambion, #2: S64346, Ambion, #3: S64347, Ambion), mouse FABP4 siRNA (#1: S62400, Ambion, #2: SASI_Mm01_00132113, Sigma-Aldrich, #3: S62398, Ambion), or mouse mitochondrial acetyl-CoA acyltransferase (ACAA2) siRNA (#1: S78883, Ambion, #2: S78884, Ambion, #3: S78885, Ambion), respectively. 3T3-L1 adipocytes were seeded in a 24-well plate and transfected with mouse CPT1a, FABP4, ACAA2, or universal negative control (SIC001, Sigma Aldrich) siRNA using Lipofectamine RNAiMAX reagent (Invitrogen) according to the manufacturer’s instructions.

### Detection of lipid droplets

3T3-L1 adipocytes were infected with *T. cruzi* for 0, 24, 36, and 48 h. *T. cruzi*-infected cells were fixed in 4% paraformaldehyde at 37°C for 30 minutes. After washing with PBS, the cells were stained with BODIPY 493/503 (Thermo Fisher Scientific, Inc.) and Hoechst 33342 (Thermo Fisher Scientific, Inc.) in the dark at room temperature for 30 minutes. After washing with PBS, the cells were mounted in a mounting medium (Prolong Gold Antifade Mountant; Life Technologies, Carlsbad, CA). Confocal images were obtained using an LSM710 confocal microscope (Carl Zeiss, Germany) and Zeiss ZEN 2010 acquisition software. A flow cytometer (FACS Canto II, BD Bioscience) was used to evaluate the fluorescence intensity of BODIPY 493/503 quantitatively.

### Free fatty acid and glycerol release assay

3T3-L1 adipocytes (2 × 10^5^) were infected with *T. cruzi* for 0, 24, 36, and 48 h. The FFA and glycerol levels in the culture supernatant were measured using the Free Fatty Acid Quantification Kit (Abcam) and glycerol cell-based assay kit (Cayman Chemical Company, Ann Arbor, MI).

### LDH cell-cytotoxicity assay

The release of LDH (lactate dehydrogenase) from dead cells was measured using the CytoTox 96 Non-Radioactive Cytotoxicity Assay Kit (Promega, Madison, WI). Treatment with 0.8% Triton X-100 (Tx-100), a non-ionic detergent, for 45 minutes was used to lyse cells and release their intracellular LDH. This procedure was also employed to establish the reference point for maximum LDH release in cell-cytotoxicity assays.

### Flow cytometric determination of cytosolic ROS, mitochondrial ROS, and mitochondrial membrane potential in adipocytes

Intracellular ROS were measured using CellROX (Molecular Probes, Eugene, OR) staining. 3T3-L1 adipocytes were incubated with CellROX (5 µM) at 37°C for 40 minutes to detect intracellular ROS 48 hours after mock or *T. cruzi* infection. Mitochondrial ROS levels were measured using MitoSOX (Molecular Probes) staining. 3T3-L1 adipocytes were incubated with the mitochondrial superoxide-specific dye MitoSOX (1 µM) at 37°C for 20 minutes to detect mitochondrial ROS 24 hours after mock or *T. cruzi* infection. The 3T3-L1 adipocytes were washed once with PBS, treated with 0.05% trypsin-EDTA, and resuspended in PBS. To distinguish between viable and dead cells, Zombie NIR Fixable Viability Kit (BioLegend, San Diego, CA) was added, incubated at 37°C for 10 minutes, washed with PBS, and then suspended in PBS again before flow cytometry. For the measurement of mitochondrial membrane potential, *T. cruzi*-infected adipocytes were stained with MitoTracker Green (250 nM, Invitrogen) and MitoTracker Red (500 nM, Invitrogen), and live cells were identified using Zombie NIR (BioLegend) and detected by flow cytometry (FACSCanto II). We consider functional mitochondria the gate containing cells stained with MitoTracker Green^high^ and MitoTracker Red^high^. The gate containing cells stained with MitoTracker Green^high^ and MitoTracker Red^low^ represents the percentage of dysfunctional mitochondria, which are mitochondria that suffered loss of membrane potential (55, 56). Data analyses and plotting were performed using FlowJo software, version 10.8.1 (Tree Star, Inc., Ashland, OR).

### Intracellular ATP assay

According to the manufacturer’s instructions, the intracellular ATP levels were analyzed using an ATP assay kit (Abcam). 3T3-L1 adipocytes (1 × 10^5^) were treated with negative control siRNA or CPT-1 siRNA, and then mock- or *T. cruzi* infection was performed. After 9 or 24 hours, the cells were lysed with ATP assay buffer and added to the wells of a 96-well plate. The absorbance at 570 nm was measured using a microplate reader (iMark, Bio-Rad Laboratories, Hercules, CA), and the ATP concentration was calculated from the ATP standard curve.

### Quantitative real-time PCR analysis

Total RNAs were extracted from cells using ISOGEN II (NIPPON GENE) and reverse-transcribed with a ReverTra Ace qPCR RT Master Mix with gDNA Remover (TOYOBO). Expression levels of iron-related genes were determined relative to that of β-actin with THUNDERBIRD SYBR qPCR Mix using a QuantStudio3, according to the manufacturer’s instructions. The sequence of PCR primers was as follows: HMOX1, 5′-GACACCTGAGGTCAAGCACAG-3′ and 5′-CCACTGCCACTGTTGCCAAC-3′; Fpn1, 5′-CCAGTCATTGGCTGTGGTTT-3′ and 5′-AGGTGGGCTCTTGTTCACAT-3′; Fth1, 5′-GTCAGCTTAGCTCTCATCAC-3′ and 5′-ACGTCTATCTGTCTATGTCTTG-3′; Fabp4, 5′-TTTGGTCACCATCCGGTCAG-3′ and 5' -TGTCGTCTGCGGTGATTTCA-3′; Gus, 5′-ATGACGAACCAGTCACC-3′, and 5′-CCTCCAGTATCTCTCTCGCAAA-3′.

### Detection of intracellular Fe^2+^

According to the manufacturer’s instructions, levels of cytoplasmic Fe2+ of adipocytes after *T. cruzi* infection were detected using FerroOrange (DOJINDO). Briefly, 3T3-L1 adipocytes were infected with *T. cruzi* for 24 h, washed three times with serum-free DMEM medium, and 1 µmol/l FerroOrange was added and incubated for 30 min at 37°C. Fluorescence intensity (Ex. 550 nm, Em. 600 nm) was measured using a fluorescence microplate reader (SPARK 10M, TECAN, Switzerland).

### Immunoblot analysis

Cells were washed once with ice-cold PBS, lysed in RIPA buffer with protease inhibitor mixture (NACALAI TESQUE, INC., Japan), and then briefly sonicated. Membranes were blocked with Blocking One (NACALAI TESQUE, INC.) for 30 min and incubated overnight at 4°C with primary Ab diluted in Can Get Signal solution 1 (TOYOBO). The membranes were then incubated for 1 hour at room temperature with HRP-conjugated secondary Abs against rabbit/mouse IgG (GE Healthcare Technologies Inc., Chicago, IL) or goat IgG (SeraCare Life Sciences, Milford, MA) diluted in Can Get Signal solution 2 (TOYOBO). Immunoreactive bands were detected using ECL blotting reagents (GE Healthcare) or EzWestLumi plus (ATTO, Japan). Densitometric analysis was performed using Image Studio Lite software (LI-COR Biosciences, Lincoln, NE).

### Statistical analysis

Results are expressed as mean ± SEM; the statistical significance of differences between the two groups was measured by an unpaired t-test. One-way or two-way ANOVA with Tukey’s multiple comparison test or Sidak’s multiple comparison test was used to analyze multiple groups. Data analyses were performed using GraphPad Prism software, version 8.4.2 (GraphPad, La Jolla, CA).

## SUPPLEMENTAL MATERIAL

Fig. S1 mbio.02180-25-s0001.tif *T. cruzi* infection induces the secretion of FABP4 from murine adipocytes.

Fig S2 mbio.02180-25-s0002.tif Effect of FAO inhibitors or FABP4 inhibitor on *T. cruzi* proliferation in the epimastigote forms.

Fig. S3 mbio.02180-25-s0003.tif Mitochondrial membrane potentials decline in murine adipocytes following *T. cruzi* infection.

ASM does not own the copyrights to Supplemental Material that may be linked to, or accessed through, an article. The authors have granted ASM a non-exclusive, world-wide license to publish the Supplemental Material files. Please contact the corresponding author directly for reuse.
