# Supplementary figures and images for: FABP4-dependent fatty acid oxidation-fueled mitochondrial ROS induces the mobilization of cellular iron and facilitates Trypanosoma cruzi proliferation in murine adipocytes
Source: mBio. 2025 Sep 8;16(10):e02180-25. doi: 10.1128/mbio.02180-25 (PMC12505895; doi:10.1128/mbio.02180-25)

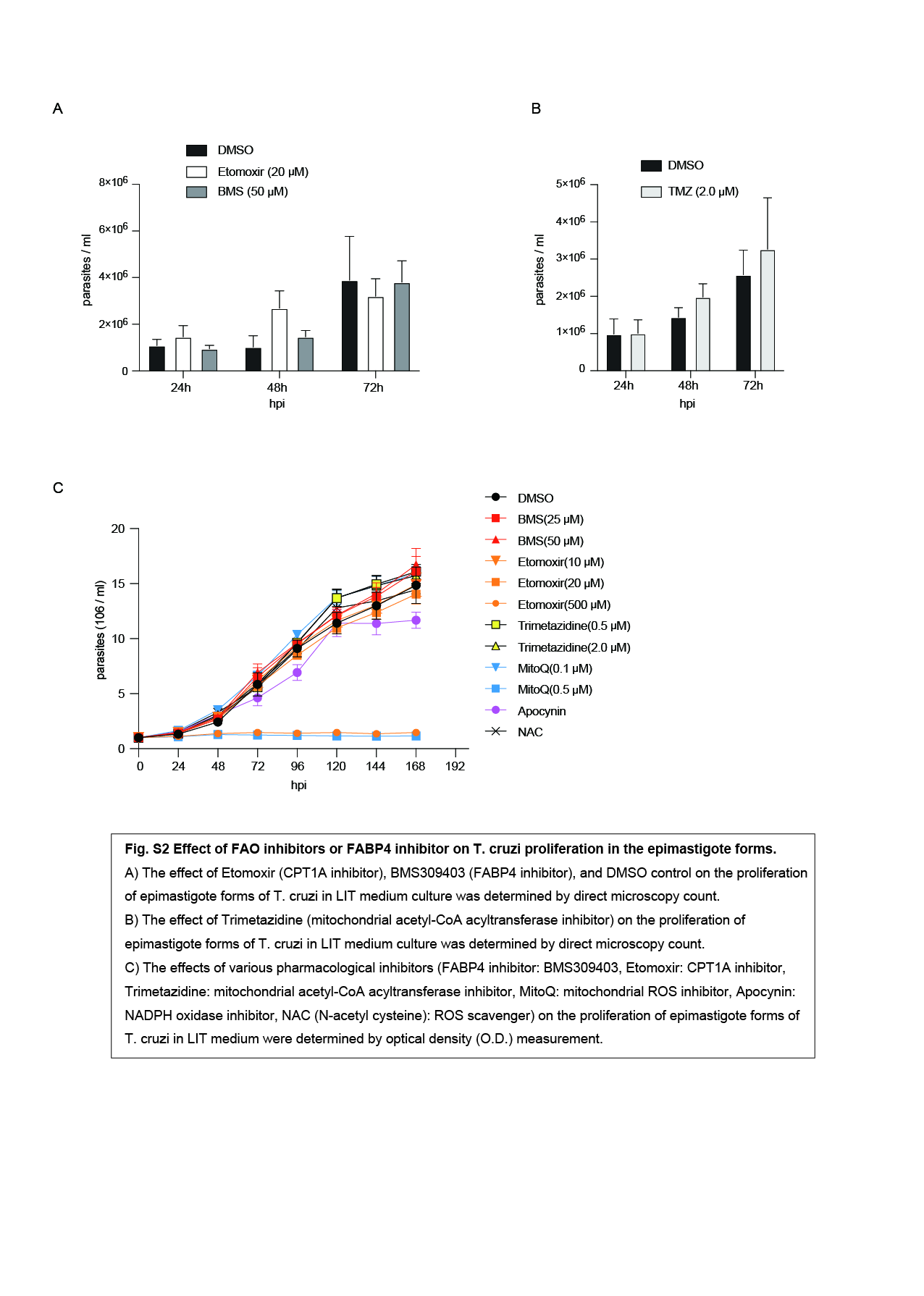

Supplement: Fig S2 — Effect of FAO inhibitors or FABP4 inhibitor on T. cruzi proliferation in the epimastigote forms. [file mbio.02180-25-s0002.tif]
